# Osteointegration of Porous Poly-ε-Caprolactone-Coated and Previtalised Magnesium Implants in Critically Sized Calvarial Bone Defects in the Mouse Model

**DOI:** 10.3390/ma11010006

**Published:** 2017-12-21

**Authors:** Michael Grau, Christian Seiler, Laura Roland, Julia Matena, Claudia Windhövel, Michael Teske, Hugo Murua Escobar, Matthias Lüpke, Hermann Seifert, Nils-Claudius Gellrich, Heinz Haferkamp, Ingo Nolte

**Affiliations:** 1 Small Animal Clinic, University of Veterinary Medicine Hannover, Foundation, D-30559 Hannover, Germany; michael.grau@tiho-hannover.de (M.G.); laura.roland@yahoo.de (L.R.); julia.matena@gmx.de (J.M.); claudia.windhoevel@tiho-hannover.de (C.W.); hugo.murua.escobar@med.uni-rostock.de (H.M.E.); 2 Division of Medicine Clinic III, Hematology, Oncology and Palliative Medicine, University of Rostock, D-18057 Rostock, Germany; 3 Institute for General Radiology and Medical Physics, University of Veterinary Medicine Hannover, Foundation, D-30173 Hannover, Germany; christian.seiler@tiho-hannover.de (C.S.); matthias.luepke@tiho-hannover.de (M.L.); hermann.seifert@tiho-hannover.de (H.S.); 4 Institute for Biomedical Engineering, Rostock University Medical Center, D-18119 Rostock, Germany; michael.teske@uni-rostock.de; 5 Clinic for Cranio-Maxillo-Facial Surgery, Hannover Medical School, D-30625 Hannover, Germany; gellrich.nils-claudius@mh-hannover.de; 6 Institut fuer Werkstoffkunde, Leibniz Universitaet Hannover, D-30823 Garbsen, Germany; haferkamp@iw.uni-hannover.de

**Keywords:** magnesium, poly-caprolactone, implant, calvarial defect, mouse, in vivo small animal imaging, micro-computed tomography, previtalisation, osteoblast, ADMSC

## Abstract

Metallic biomaterials are widely used in maxillofacial surgery. While titanium is presumed to be the gold standard, magnesium-based implants are a current topic of interest and investigation due to their biocompatible, osteoconductive and degradable properties. This study investigates the effects of poly-ε-caprolactone-coated and previtalised magnesium implants on osteointegration within murine calvarial bone defects: After setting a 3 mm × 3 mm defect into the calvaria of 40 BALB/c mice the animals were treated with poly-ε-caprolactone-coated porous magnesium implants (without previtalisation or previtalised with either osteoblasts or adipose derived mesenchymal stem cells), porous Ti6Al4V implants or without any implant. To evaluate bone formation and implant degradation, micro-computertomographic scans were performed at day 0, 28, 56 and 84 after surgery. Additionally, histological thin sections were prepared and evaluated histomorphometrically. The outcomes revealed no significant differences within the differently treated groups regarding bone formation and the amount of osteoid. While the implant degradation resulted in implant shifting, both implant geometry and previtalisation appeared to have positive effects on vascularisation. Although adjustments in degradation behaviour and implant fixation are indicated, this study still considers magnesium as a promising alternative to titanium-based implants in maxillofacial surgery in future.

## 1. Introduction

In maxillofacial surgery, the treatment of critically sized bone defects whether of neoplastic, traumatic or congenital origin, is still challenging due to insufficient vascularisation and therefore inadequate bone regeneration [1,2]. Originally, the term critically sized defect was defined as the smallest size of a defect that would not heal within the lifetime of the animal [3]. Several studies stated defects with 5 mm in diameter to be critically sized for calvaria of adult mice [4,5]. Due to convenience issues in animal research a critical defect often alternatively refers to a size that is not able to heal throughout the duration of the experiment [5,6]. Since different studies have shown that defects of 3 mm in diameter set within the calvaria of mice did not heal within a period of 12 weeks [7,8,9], the same criteria were chosen for this study both time and dimension wise. For closing critically sized defects, autografts harvested from intact bone are commonly used. However, this treatment is limited to a certain degree in terms of accessible bone material and also requires an additional surgical intervention, which elevates the patient’s morbidity and often remains painful after surgery [10,11,12,13]. In order to avoid such challenges the usage of metallic biomaterials in form of implants is required. Titanium and its alloys are often used owing to their high biocompatibility and corrosion resistance but involve the risk of implant loosening as a result of stress shielding due to the discrepancy in Young’s modulus to cortical bone [14,15,16].

Magnesium and its alloys might conquer these difficulties since their mechanical properties are much more bone-like. Namely, its Young’s modulus is closer to that of osseous tissue and due to its biodegradability it enables ingrowing bone to slowly take over the implant’s space and stabilising function [17,18,19,20,21]. In recent years, magnesium has been proven to possess high biocompatibility in various studies [19,22,23]. However, the main challenge using magnesium-based implants is the gas formation due to the material’s degradation process, leading to displacement of surrounding tissues and a decrease in implant-bone contact area [24,25]. A possible way to contain the initial corrosion process is to create a coating around the implant in order to shield the metallic core against the surrounding tissue fluid [26]. In the following sections the combination of a metallic basic structure and a polymeric coating is referred to as a hybrid implant. For this study we decided to coat the implants with the synthetic resorbable polymer poly-ε-caprolactone (PCL) since it is an established biomaterial, while at the same time showing a lowering effect on the corrosion rate of magnesium implants [27,28,29,30,31]. Namely, several studies prove that PCL-coating of magnesium implants leads to an increased initial corrosion resistance resulting in a decreased release of gas and a lowering of the implants’ degradation rate which ultimately improves cytocompatibility for osteogenic cells [30,32,33]. In addition to the natural osteoconductive properties of magnesium, this quality can even become enhanced by creating a porous implant geometry, which allows newly formed bone tissue to grow inside the pores. Within this study, scaffold-shaped implants with a pore size of 600 µm were created using the additive manufacturing process of Selective Laser Melting (SLM^®^, Lübeck, Germany) since pore sizes of 20 to 1500 µm were reported to enable an optimal osteoblast activity and therefore a sufficient osteointegration of the implant [34]. In order to enhance the latter, even further implants can be previtalised with adipose-derived mesenchymal stem cells (ADMSCs), which have become a focus of research within recent years [35]. Since they are reported to be able to differentiate into osteoblasts, they are stated to have a positive effect on bone regeneration by producing new osseous tissue [36,37,38]. As a high amount of ADMSCs can be isolated fast and in a minimally invasive manner from the acceptor’s own adipose tissue [39,40] they may be preferable over already differentiated osteoblasts for implant previtalisation.

Overall, this study deals with finding an implant material that is able to act as an alternative treatment for critically sized calvarial defects to hitherto merchantable titanium implants. Namely, it describes the local interaction of the surrounding bone tissue and the inserted hybrid implant. Therefore, the implants were tested within an in-vivo mouse model against uncoated titanium implants of the same geometry. As a negative control, mice without any implant were observed. In order to not consciously expose the animals to excessive gas production and possible side effects uncoated magnesium scaffolds were not used as a negative control. The uncontrolled gas production can lead to subcutaneous gas pockets, tissue necrosis, infections, alkaline poisoning and sudden death due to gas-induced embolism as stated by several studies [41,42,43,44]. To test the influence of previtalisation the hybrid implants were either populated with osteoblasts, ADMSCs or left blank. After inserting the implants within a previously set critically sized calvarial defect, bone ingrowth and implant degradation were observed via µCT scans for three months. Additionally, histomorphometrical analyses were performed to gain a closer view of the histological changes within the defect region. An enhanced amount of bone ingrowth in the hybrid implants due to the material’s osteoconductivity was expected. Also, an even higher osteointegration was suggested for the previtalised implants since bone regeneration could begin not just at the defect periphery but also at the implant itself. Since the PCL-coating was created to reduce the corrosion rate of magnesium, high bone-implant contacts as well as a balanced substitution rate (defined as the quotient of implant degradation and bone ingrowth) were anticipated. 

## 2. Experimental Section

### 2.1. Selective Laser Melting of Titanium and Magnesium Implants

Using the SLM technology, three-dimensional porous titanium scaffolds were manufactured by SLM^®^ Solutions GmbH, Lübeck, Germany. Thereby, an SLM^®^ 280HL Selective Laser Melting machine system was utilised in order to transform Ti6Al4V powder into solid scaffolds. The magnesium implants with identical geometry were manufactured from ATOULTRA 325 pure magnesium powder (SFM SA, Martigny, Switzerland) using an SLM^®^ 125HL machine system. All implants were post-treated using a chemical deburring process in order to create a smooth implant surface [45].

**Figure 1:**
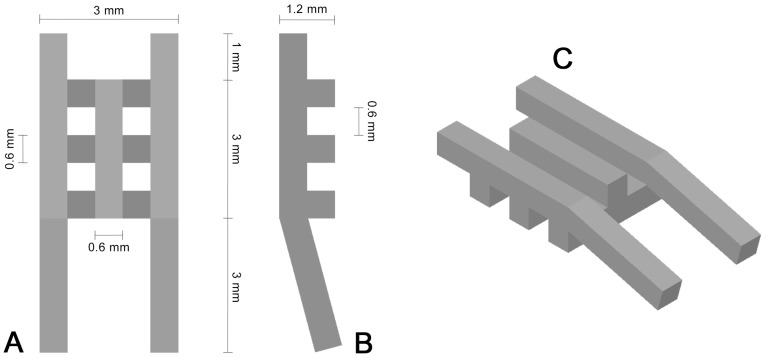
Schematic top view (**A**); side view (**B**) and three-dimensional view (**C**) of the scaffold geometry.

The generated implants (geometry shown in Figure 1) consisted of a 3 mm × 3 mm × 1.2 mm basic body with a pore size and strut width of 600 µm. To prevent the implants from sinking into the bone defect, the two outer struts of the upper implant layer were elongated at both sides 1 or 3 mm, respectively. The longer elongated struts were bent at an angle of 15 degrees in order to ensure a proper fixation to the animal’s skull.

### 2.2. PCL-Coating of Magnesium Implants

The magnesium scaffolds were plunged 8 times into 0.8 wt. % chloroformic (p.a. quality, J. T. Baker, Deventeer, The Netherlands) PCL (M_n_ = 80.000 g/mol, Cappa 6800, Perstorp UK Limited, Warrington, UK) solution using a custom-designed sample holderwith fork type tongue rings (1.5–2.5 mm cross section) to fix the samples mechanically between both forks. Between each dipping process the scaffolds were dried for 10 min at 23 ± 2 °C to allow the chloroform to evaporate. After the final dipping step the scaffolds were dried at room temperature for 24 h. For the following step the implants were carefully removed from the sample holders and both uncoated contact points were covered with 3 μL of a 5 wt % polymer solution of PCL. Therefore, the concentration was optimised, allowing a fast bonding with the existing coating without changes in coating thickness. Afterwards, the coatings were dried for 20 min at room temperature. The final drying process was performed in a vacuum cabinet drier for seven days at 40 °C and 40 mbar.

### 2.3. Murine Osteoblast Isolation

Calvariae of ten C57Bl6 mice were grinded into small pieces which were then digested five times, each time for 10 min at 37 °C in sterile Hank’s medium (HBSS, PAA Laboratories GmbH, Pasching, Austria) containing 5 mL of 200 U/mL collagenase II (Cell Systems, Troisdorf, Germany). After each digestion step the supernatant containing diluted cells was collected and substituted with new digestion medium, resulting in a pooled cell solution of all five digestion steps. After centrifuging the solution for 7 min at 1200 RPM at room temperature the cell pellet was washed twice with Dulbecco’s Modified Eagle Medium (DMEM) (Biochrom AG, Berlin, Germany) containing 10% foetal calf serum (FCS, Biochrom AG, Berlin, Germany), 20 mM Hepes, 1000 IU/mL penicillin and 0.1 mg/mL streptomycin (both PAA, Coelbe, Germany). After resuspending the pellet the cells were seeded onto T25 tissue culture flasks (TPP, Trasadingen, Switzerland) filled with 5 mL DMEM containing 10% FCS and incubated at 37 °C and 5% CO_2_. The culture medium was changed twice a week and cell populations were split at 80% confluence. 

### 2.4. Murine ADMSC Isolation

Inguinal fat tissue was isolated from BALB/c mice and stored in sterile Hank’s medium until further processing. Afterwards, the tissue was washed three times with phosphate buffered saline (PBS, Biochrom AG, Berlin, Germany), separated from adhering connectiveand vascular tissue and shredded with a scalpel. The resulting material was then added to a tube filled with 5 mL 0.026% Collagenase solution and incubated for 1 h at 37 °C while being rotated within a MACmix™ Tube Rotator (Milteny Biotec GmbH, Bergisch Gladbach, Germany). After incubation the resulting collagenase-cell-solution was added to 5 mL DMEM containing 10% FCS and centrifuged for 10 min at 1000 RPM at room temperature. Before resuspending the cell pelletin 1 mL culture medium the supernatant was removed. The resulting collagenase-free cell solution was then added to T25 tissue culture flasks (TPP, Trasadingen, Switzerland) filled with 5 mL DMEM containing 10% FCS and incubated at 37 °C and 5% CO_2_. The culture medium was changed twice a week and cell populations were split at 80% confluence.

### 2.5. Implant Previtalisation

For previtalisation the implants were inserted into the wells of a flat bottomed 96 well plate filled with 150 µL DMEM containing 10% FCS. After trypsinating murine osteoblasts (P 1) and murine ADMSCs (P 1) from culture flasks the upfacing surfaces of each of the eight hybrid implantswere previtalised with either 25 × 10^3^ osteoblasts or the same amount of ADMSCs according to Table 1. After an attachment time of one hour under cell culture conditions (37 °C, 5% CO_2_) the implants were flipped upside down, the procedure then being repeated on the other side of the scaffold. After an additional attachment time of one hour the implants were finally stored within the wells of a 6-well plate filled with 5 mL DMEM containing 10% FCS and kept at 37 °C and 5% CO_2_ until the following morning (day of surgery). The attachment times were set at one hour to avoid any negative effects arising from magnesium corrosion products on cellular vitality.

### 2.6. Animal Grouping and Treatment

A total number of 40 eight-week-old female BALB/c mice (Charles River Laboratories, Wilmington, MA, USA) were allocated to five experimental groups of eight according to Table 1 and kept in groups of four (two cages per experimental group) in cages bedded withArbocel^®^ comfort natural bedding material (Altromin GmbH, Lage, Germany). Maintenance diet pellets (Altromin GmbH) as well as water via polycarbonate bottles were offered ad libitum during the whole experimental period. The cages were stored in a climatic chamber (Tecniplast S.p.A., Buguggiate, Varese, Italy) in a 12 h day-night rhythm, checking temperature and humidity each day using a digital thermohygrometer (TFA Dostmann GmbH, Wertheim, Germany). Before surgery the mice were kept for four weeks, handling them each day to ensure a proper acclimatisation and an overall low stress level.

After surgery the animals were treated analgesically (Carprofen 5 mg/kg s.c. and Tramadol 1 mg/mL drinking water p.o. for three days) and weighed constantly in order to monitor changes in health and eating behaviour. One mouse in group 3, two mice in group 4 and three mice in group 5 rejected their implant before the second µCT scan. The resulting wounds healed properly within a few days under analgesic treatment (Tramadol 1 mg/mL drinking water for three days) without any signs of infectious inflammation and did not affect the animal’s eating, drinking or comfort behaviour. Therefore, no intervention in form of early euthanasia was indicated.

### 2.7. Surgical Methods

After anaesthetising with ketamin (100 mg/kg i.p.) and xylazin (4–12 mg/kg i.p.) the skin above the implantation region was depilated, washed and disinfected. To avoid intraoperative hypothermia the animal was layed on a MouseMonitor™ S warming plate (Indus Instruments, Webster, TX, USA) which ensured a constant body temperature. The entire surgery was performed under sterile conditions using a Universal S3 surgery microscope (Carl Zeiss AG, Oberkochen, Germany). The skin was cut and retracted in order to gain a proper view of the osseous calvarium. Using a diamond milling head on a MICROMOT 50/E milling machine (Proxxon GmbH, Föhren, Germany), an approximately 3 mm × 3 mm square bone platelet was milled out, while preserving surrounding blood vessels and underlying meninges. Within this process the operating area was constantly cleared and cooled with sterile saline solution in order to ensure a clear view and to avoid any heat caused by the milling process. After removing the bone platelet a magnesium or titanium implant was inserted in the created defect according to the animal group number as described in 2.6. To avoid both damage to the meninges due to the implant sinking into the defect and implant shifting during the experiment, the elongated struts were attached to the opposite side of the calvarium using an ethyl-2-cyanoacrylate-based tissue glue (EPIGLU^®^, Meyer-Haake Medical Innovations, Ober-Mörlen, Germany). Thereafter, the skin was sutured with resorbable sewing material. In the case of control group 1 the skin was closed in a similar manner but without inserting any implant. After the following first µCT scan the mice were treated analgesically with Carprofen (5 mg/kg s.c.) and received a subcutaneous saline infusion while lying under an infrared lamp to ensure a safe and proper recovery.

### 2.8. µCT Scans and Data Analysis

All µCT scans were performed using an XtremeCT (Scanco Medical AG, Brüttisellen, Switzerland) with a fixed tube voltage of 60 kV. The resolution and integration time were set at 41 µm and 700 ms, respectively. In order to reduce anaesthesia time especially at the first scan right after surgery, only a small part of approximately 9 mm of the skull was scanned. Therefore, each scan lasted about 15 min and contained 220 slices.

**Figure 2:**
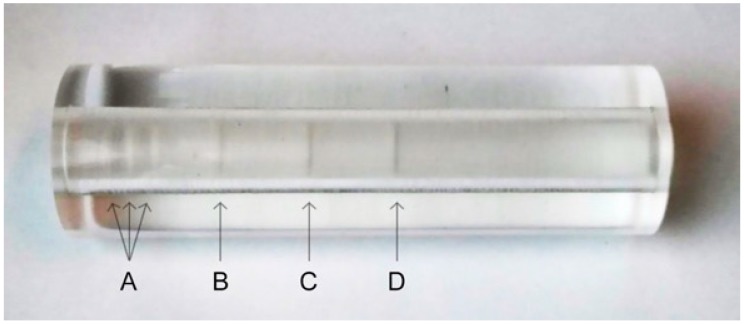
Two-piece acrylic glass mouse phantom (diameter: 3 cm; length: 10 cm). The inner pin (diameter: 1 cm) contains six cavities for one TLD each for measuring the exposure during the µCT scans. Three cavities were placed within 1 cm (head region of the mouse; (**A**) and the others at a distance of 2 cm (lung; (**B**)); 3.5 cm (kidney; (**C**)) and 5 cm (abdomen; (**D**)) from the first cavity.

To evaluate the absorbed dose per mouse during one µCT scan a cylindric acrylic glass body (Figure 2) with small cavities for lithium-fluoride thermoluminescent dosimeter (TLD) (TLD-100H, Thermo Fisher Scientific, Waltham, MA, USA) was used as a mouse phantom. The phantom was scanned with the same parameters that were used for the actual experimental scans.

**Figure 3:**
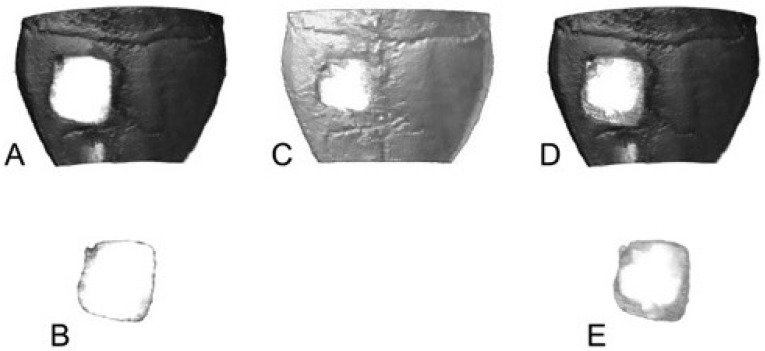
µCT scans of the skull. Dorsal view at the calvaria of a mouse from control group 1 at day 0 (**A**) and the (cut out ROI (**B**); Same view at day 28 without (**C**) and with overlaid first scan (**D**) resulting in the ROI for day 28 (**E**)).

The resulting scan data were evaluated using the Amira 3D visualisation and analysis software (version 5.5.0, FEI Visualization Sciences Group, Hillsboro, OR, USA). Primarily, the threshold was set at 450 Hounsfield Units (HU) to separate soft from bony tissue. Since they showed similar HU, both magnesium implants and bone tissue were detected within the same threshold range. Nevertheless, both structures were determined separately due to the characteristic implant geometry. Next, the bone defect of the first scan (day 0, Figure 3A) was cut out manually so only a fine rim of bone remained around the defect (Figure 3B). This shape was then used as a blueprint and projected on the skulls in scans of day 28 (Figure 3C,D), 56 and 84 respectively to cut out the exact same region of interest (ROI, starting point of defect healing, Figure 3E). Afterwards, the volume of the bone around the defects was calculated automatically.

For examining the contralateral side, acuboid ROI of 3.7 mm × 3.7 mm × 2.0 mm was placed in the middle of the left calvaria side and analysed regarding bone volume in a similar manner to the treated side. Since the titanium implants unfortunately caused massive artefacts in the µCT scans there was no possibility of analysing the bone ingrowth in control group 2.

Regarding the magnesium implant degradation, the analysing process followed the same steps but used another ROI around the scaffold. For each group, the average values for both defect healing and implant degradation were determined for every scanning day.

### 2.9. Preparation of Histological Thin Sections

After the final µCT scan at day 84 the mice were euthanised in anaesthesia using 0.15 mL pentobarbital i.p. and decapitated after ensuring death by heart beat absence. Before commencing the dehydration procedure for technovit embedding, the heads were stored in 4% formaldehyde. The dehydration process was performed using decreasing alcohol concentrations followed by embedding in technovit 9100 Neu. Resulting technovit blocks were cut into thin sections using a diamond band saw (EXAKT Advanced Technologies GmbH, Norderstedt, Germany). Afterwards, the thin sections were grinded and polished by a lap-grinder grinding machine (patho-service GmbH, Oststeinbeck, Germany) in order to achieve sections of about 30 µm thickness. To achieve a high contrast between mineralised bone tissue and newly formed osteoid, trichrome-masson-goldner staining was performed. Afterwards, the stained and Eukitt-covered thin sections were photographed with the computer-controlled microscope Axio Imager.Z1 (Carl Zeiss AG, Oberkochen, Germany) using the AxioVision SE64 software (version 4.9.1.0; Carl Zeiss AG, Oberkochen, Germany). All examined images can be found in the Supplementary Materials (Figures S2–S124).

### 2.10. Histomorphometrical Analysis

**Figure 4:**
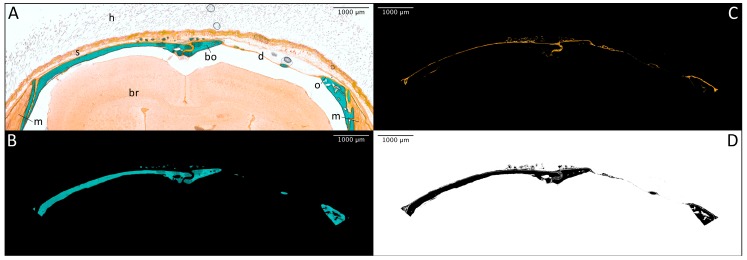
Laterolateral thin section of a mouse without an implant after trichrome-masson-goldner staining ((**A**); bo = mineralised bone tissue, br = brain, d = defect, h = hair, m = muscle tissue, o = osteoid seam, s = skin; 10-fold magnification); mineralised bone tissue (turquoise) after thresholding (**B**); osteoid (orange) after thresholding and subtracting fibrotic and muscular tissue (**C**); double-thresholded total bone area (black = mineralised bone tissue + osteoid) ready for analysis (**D**).

Using the thresholding function of ImageJ picture analysis software, the images (Figure 4A) were separated into different colour ranges. While mineralised bone tissue was stained in bright turquoise and therefore easily detectable, the osteoid seams shared their orange colouration with fibrotic capsule strings as well as muscular and connective tissue of the overlaying skin. Since only newly formed bone tissue should be analysed, the unwanted structures were identified due to their characteristic morphological and cellular structure and deleted before analysis. The resulting single images for mineralised (Figure 4B) and unmineralised (Figure 4C) bone tissue were then analysed separately and also combined (Figure 4D) regarding their surface area using the program’s thresholding and measuring function. Therefore, the total bone area per slide was determined as well as the percentage proportion of osteoid.

As a parameter of defect regeneration the total amount of intraosteal blood vessels was counted for each section. This and all previously described measurements were performed for both the left and the right side of the skull in order to compare the defect (and possibly implant) treated right side with the untreated left side. Since the implants were attached to the left side of the skull possible influences of this anchoring could also be analysed.

**Figure 5:**
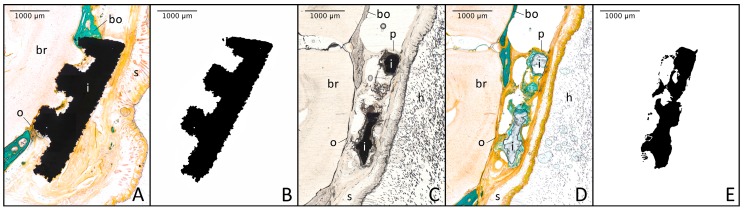
Laterolateral thin section of a mouse treated with an uncoated titanium implant (**A**) or an PCL-coated magnesium implant (**C**,**D**) before (**C**) or after trichrome-masson-goldner staining ((**A**,**D**); bo = mineralised bone tissue, br = brain, h = hair, i = implant in the defect, o = osteoid, p = PCL, s = skin; 10-fold magnification); thresholded uncoated titanium implant (**B**) or manually reconstructed PCL-coated magnesium implant (**E**) ready for analysis.

In order to evaluate the implant bone interaction, the titanium implants were thresholded (Figure 5B) and their surface length was determined. Unfortunately, the staining process caused the dissolution of the magnesium implants, but by using pre-staining photographs (Figure 5C) the implants could be reconstructed and analysed in a manner similar to titanium ones (Figure 5E). After measuring the implant surface length, the single pictures for mineralised bone tissue, osteoid and implant material were overlayed and the length of the contact surfaces was measured, resulting in the percentage values of implant bone contact.

### 2.11. Statistical Analysis

Statistical analyses of data were performed using SAS^®^ software (version 9.3, SAS Institute Inc., Cary, NC, USA). Type I error was set at 5% so p-values below 0.05 were considered statistically significant. Since the entire data were normally distributed the Ryan-Einot-Gabriel-Welsh Multiple Range test and the two-sample t-test were performed to detect significant differences between the experimental groups. Regarding the comparison of the different times of observation within each group, analyses of variance (ANOVA) for repeated measurements were performed. In this regard only successive observation times were compared to one another. In general, outliers were defined as values lying more than 1.5 times the interquatile range away from the upper or the lower quartile.

## 3. Results

The median values as well as the standard deviations for bone volume, implant degradation, bone area, osteoid and blood vessel measurement can be found in Supplementary Materials (Table S1).

### 3.1. Evaluation of µCT Scans

#### 3.1.1. Radiation Exposure

The evaluation of the TLDs showed that each mouse was exposed to an absorbed dose of about 30 mGy per scan at the head region, 2.4 mGy at the lung, 0.7 mGy at the kidneys and 0.4 mGy at the abdomen, respectively.

#### 3.1.2. Bone Volume

**Figure 6:**
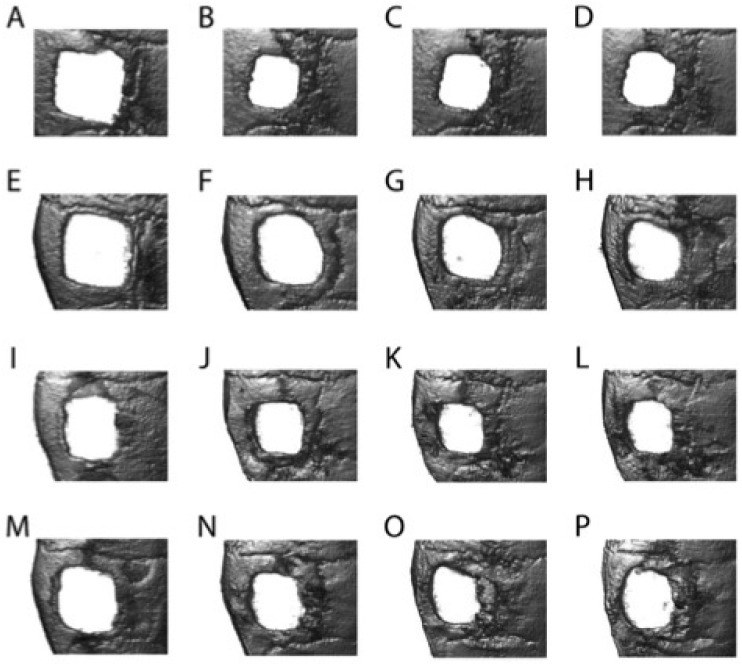
Top view of representative µCT scans of the defect of control group 1 (without implant; (**A**–**D**)) as well as group 3 (hybrid implant; (**E**–**H**)), group 4 (hybrid implant + murine osteoblasts; (**I**–**L**)) and group 5 (hybrid implant + mADMSCs; (**M**–**P**)) at days 0 (**A**,**E**,**I**,**M**), 28 (**B**,**F**,**J**,**N**), 56 (**C**,**G**,**K**,**O**) and 84 (**D**,**H**,**L**,**P**). For any other but the control group the implant was cut out in order to have a proper view of the defect.

Judging from the µCT scans for each measurement day, semiquantitatively the largest increase in bone volume took place between days 0 and 28 for each experimental group, while within the subsequent scans the defects of each group appeared to remain stable. Figure 6 exemplarily illustrates this development in bone volume increase for control group 1 (without implant) as well as for the groups 3 to 5. Since titanium caused massive artefacts in the µCT images (see Supplementary Materials Figure S1 for an exemplary image) demarcation of the implant to the defect and therefore an observation of the defect closure were not possible within this experiment.

**Figure 7:**
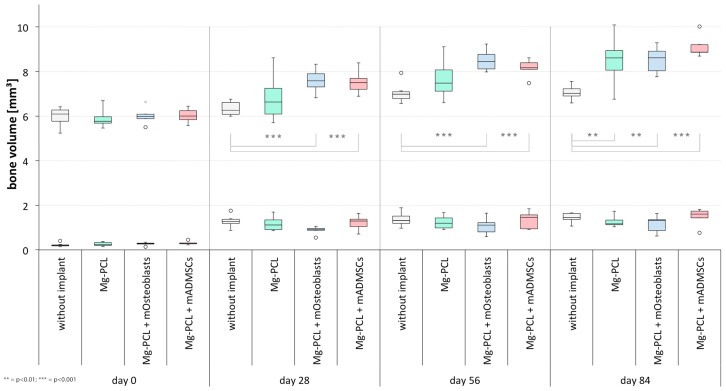
Comparative presentation of the formation of bone volume on the defect treated right side (lower row) and untreated left side (upper row) between the experimental groups at days 0, 28, 56 and 84 (** = *p* < 0.01; *** = *p* < 0.001; whiskers = minimal and maximal values; horizontal line = median; circles = outliers).

**Figure 8:**
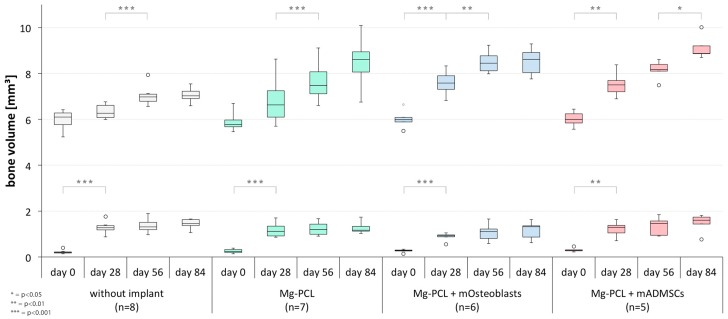
Comparative presentation of the formation of bone volume on the defect treated right side (lower row) and untreated left side (upper row) between the different measurement times within each experimental group (*= *p* < 0.05; ** = *p* < 0.01; *** = *p* < 0.001; whiskers = minimal and maximal values; horizontal line = median; circles = outliers).

At day 0 there were no significant differences in bone volume between the different groups on the treated skull side, this emphasising the high amount of accuracy put into creating the surgical defect (Figure 7, lower row). All groups showed a significant increase in bone volume between days 0 and 28 (Figure 8, lower row; without implant: *p* < 0.0001; Mg-PCL: *p* = 0.0003; Mg-PCL + mOsteoblasts: *p* < 0.0001; Mg-PCL + mADMSCs: *p* = 0.0033). Neither between the subsequent measurement days for each experimental group (Figure 8, lower row) nor between the groups at each measured time (Figure 7, lower row) could significant differences in bone volume formation be detected.

However, on the untreated left side significant differences in bone volume could be revealed between control group 1 (only defect) and both Mg-PCL treated groups previtalised with mOsteoblasts or mADMSCs at days 28 (Mg-PCL + mOsteoblasts: *p* = 0.0001; Mg-PCL + mADMSCs *p* = 0.0007), 56 (Mg-PCL + mOsteoblasts: *p* < 0.0001; Mg-PCL + mADMSCs *p* = 0.0007) and 84 (both *p* < 0.0001). At day 84 there was also a significant difference in bone volume between control group 1 and the non-previtalised MG-PCL group (*p* = 0.0031; Figure 7 upper row).

Regarding the bone volume development between the different measurement times (Figure 8, upper row) significant differences could be detected between days 28 and 56 within control group 1 (*p* = 0.0004) as well as within the non-previtalised Mg-PCL group (*p* = 0.0003) and the Mg-PCL group previtalised with mOsteoblasts (*p* = 0.0064). Additionally, there were significant differences between days 0 and 28 for both previtalised Mg-PCL groups (mOsteoblasts: *p* = 0.0009; mADMSCs: *p* = 0.0069). Within the Mg-PCL group previtalised with mADMSCs also a significant difference in bone volume formation could be detected between days 56 and 84 (*p* = 0.0338).

#### 3.1.3. Implant Degradation

**Figure 9:**
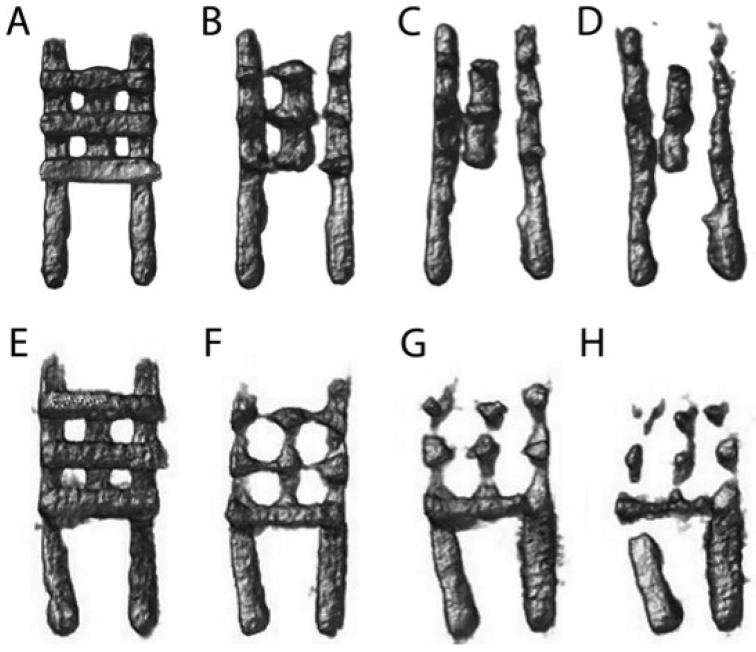

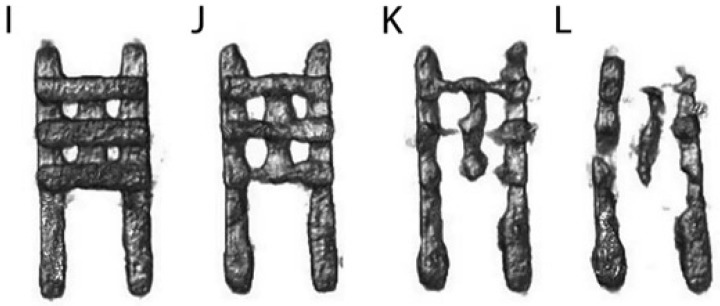
Top view of representative µCT scans of Mg-PCL implants without previtalisation (**A**–**D**), or previtalised with either murine osteoblasts (**E**–**H**) or mADMSCs (**I**–**L**) at days 0 (**A**,**E**,**I**), 28 (**B**,**F**,**J**), 56 (**C**,**G**,**K**) and 84 (**D**,**H**,**L**).

Judging from the µCT scans for each measurement day, semiquantitatively the magnesium implant volume decreased consistently during the 84 days of observation (Figure 9). The degradation process pertained at first the bars facing the defect (cf. Figure 5C–E). Figure 9 exemplarily illustrates this development in implant degradation for non-previtalised magnesium implants.

**Figure 10:**
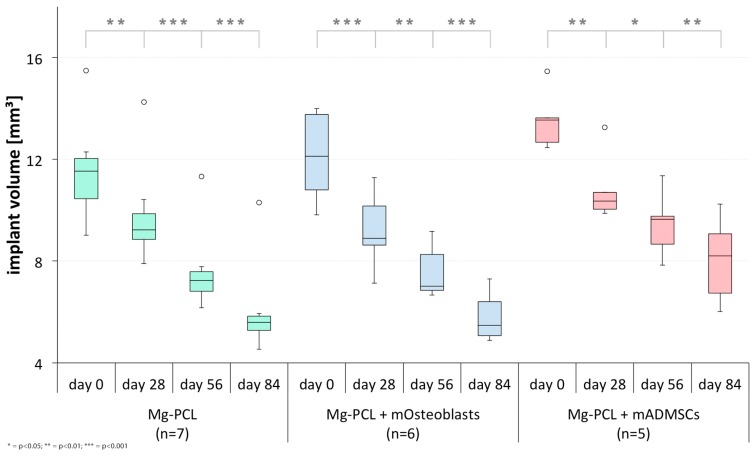
Comparative presentation of the implant degradation between the different measurement times within each magnesium implant-treated group (* = *p* < 0.05; ** = *p* < 0.01; *** = *p* < 0.001; whiskers = minimal and maximal values; horizontal line = median; circles = outliers).

Within each group a significant decrease in implant volume was detected from scan to scan (Figure 10) (Mg-PCL: days 0 to 28: *p* = 0.0052, days 28 to 56: *p* < 0.0001, days 56 to 84: *p* = 0.0004; Mg-PCL + mOsteoblasts: days 0 to 28: *p* = 0.0004, days 28 to 56: *p* = 0.0010, days 56 to 84: *p* < 0.0001; Mg-PCL + mADMSCs: days 0 to 28: *p* = 0.0011, days 28 to 56: *p* = 0.0114, days 56 to 84: *p* = 0.0036). However, between the groups there was no significant difference in implant degradation.

#### 3.1.4. Substitution Index

**Figure 11:**
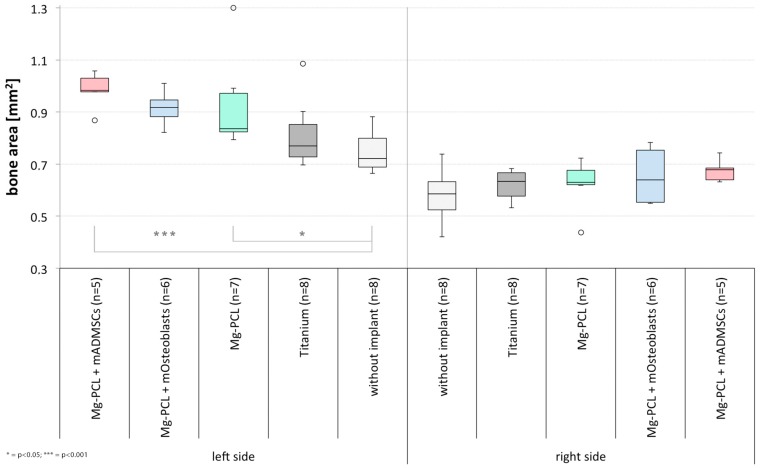
Measured bone area on slides of mice treated without an implant, with uncoated titanium implants, non-previtalised Mg-PCL implants and Mg-PCL implants previtalised with either mOsteoblasts or mADMSCs for both skull halves (* = *p* < 0.05; *** = *p* < 0.001; whiskers = minimal and maximal values; horizontal line = median; circles = outliers).

The median substitution index (quotient of total implant volume decrease and total bone volume increase) amounted to 4.670 for the Mg-PCL implant treated group (*n* = 7; standard deviation = sd = 1.057), 6.156 for the Mg-PCL implant treated group previtalised with mOsteoblasts (*n* = 6; sd = 0.908) and 6.286 for the Mg-PCL implant treated group previtalised with mADMSCs (*n* = 5; sd = 3.908; Figure 11).

### 3.2. Histomorphometry

#### 3.2.1. Bone Area

On the possibly implant-treated right side of the skull no significant differences between the experimental groups could be observed (Figure 11). However, on the untreated left half significantly higher amounts of bone could be found in the groups treated with non-previtalised Mg-PCL implants or the ones previtalised with mADMSCs in comparison to control group 1 (Mg-PCL: *p* = 0.0181; Mg-PCL + mADMSCs: *p* = 0.0002).

#### 3.2.2. Proportion of Osteoid on Total Bone Tissue

**Figure 12:**
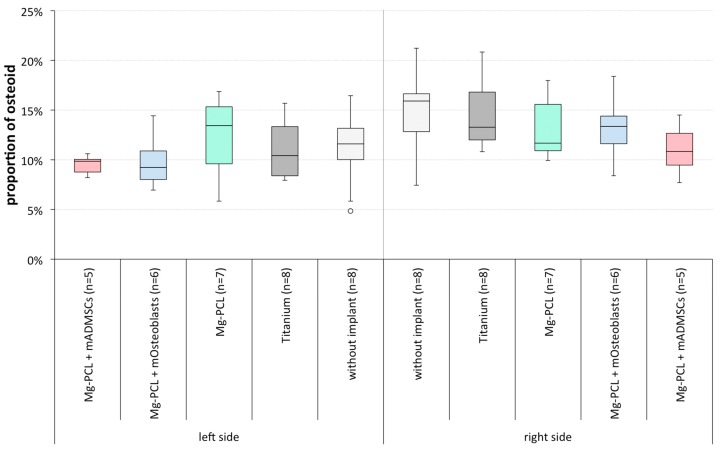
Proportion of osteoid on the total bone tissue (%) on slides of mice treated without an implant, with uncoated titanium implants, non-previtalised Mg-PCL implants and Mg-PCL implants previtalised with either mOsteoblasts or mADMSCs for both skull halves(whiskers = minimal and maximal values; horizontal line = median; circles = outliers).

Neither on the left nor on the right skull half could significant differences between the tested groups be observed regarding the amount of newly produced bone tissue (Figure 12). When comparing the values for both sides within each group, also no significant differences could be detected.

#### 3.2.3. Intraosteal Blood Vessels

**Figure 13:**
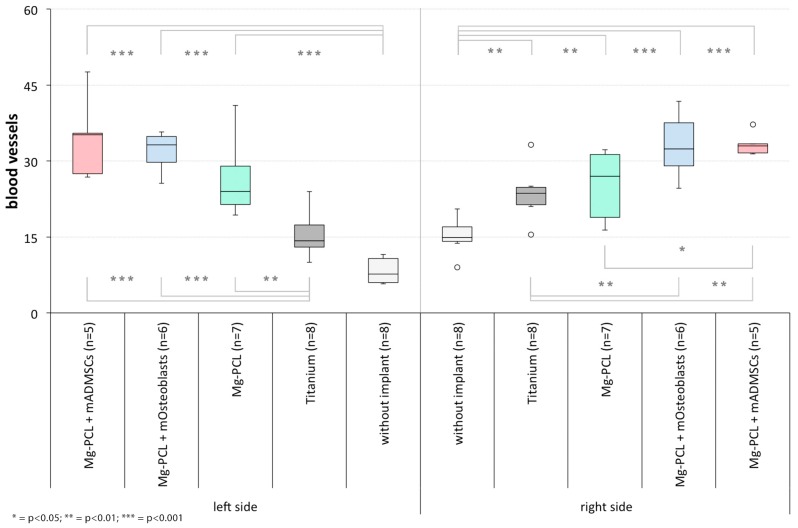
Counted intraosteal blood vessels on slides of mice treated without an implant, with uncoated titanium implants, non-previtalised Mg-PCL implants and Mg-PCL implants previtalised with either mOsteoblasts or mADMSCs for both skull halves(* = *p* < 0.05; ** = *p* < 0.01; *** = *p* < 0.001; whiskers = minimal and maximal values; horizontal line = median; circles = outliers).

On both the treated and untreated skull half a significant increase in the number of blood vessels could be found for each implant-treated group (regardless of the implant material, coating or previtalisation status) in comparison to control group 1 (without implant) apart from titanium on the left side (Figure 13; left side: Mg-PCL: *p* < 0.0001, Mg-PCL + mOsteoblasts: *p* < 0.0001, Mg-PCL + mADMSCs: *p* < 0.0001; right side: Ti: *p* = 0.0015, Mg-PCL: *p* = 0.0087, Mg-PCL + mOsteoblasts: *p* < 0.0001, Mg-PCL + mADMSCs: *p* < 0.0001). Also, significantly higher values could be observed for both previtalised Mg-PCL-implant-treated groups in comparison to control group 2 (Titanium) on both sides (left side: Mg-PCL + mOsteoblasts: *p* < 0.0001, Mg-PCL + mADMSCs: *p* = 0.0002; right side: Mg-PCL + mOsteoblasts: *p* = 0.0089, Mg-PCL + mADMSCs: *p* = 0.0018) and also for the non-previtalised Mg-PCL-treated group on the left skull half (*p* = 0.0041). Additionally, a significant decrease in blood vessels was shown for the Mg-PCL-treated group previtalised with mADMSCs in comparison to the non-previtalised Mg-PCL-treated group on the right skull half (*p* = 0.0216).

**Figure 14:**
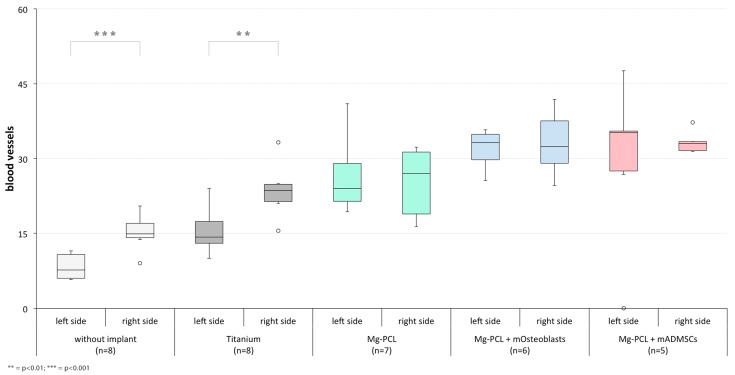
Comparison of counted intraosteal blood vessels of both skull sides within each experimental group (without an implant, with uncoated titanium implants, non-previtalised Mg-PCL implants and Mg-PCL implants previtalised with either mOsteoblasts or mADMSCs; ** = *p* < 0.01; *** = *p* < 0.001; whiskers = minimal and maximal values; horizontal line = median; circles = outliers).

When comparing the counted blood vessels on both skull sides with each other within each experimental group, significant differences could only be detected for both control groups (Figure 14; without implant: *p* = 0.0003; Ti: *p* = 0.0047).

#### 3.2.4. Implant Bone Contact

**Figure 15:**
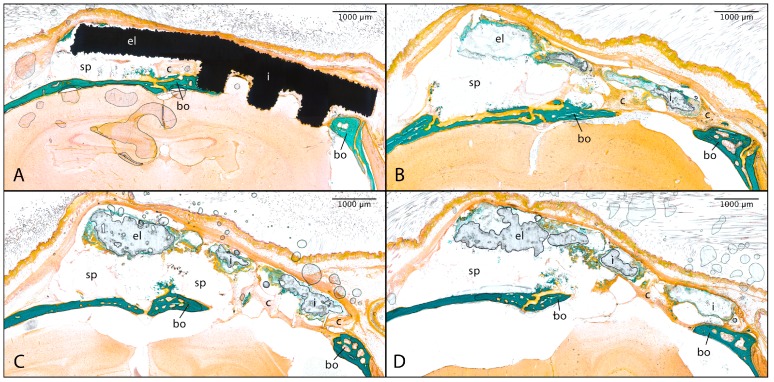
Representative histological images of the implant bone contact after 84 days for mice treated with either uncoated Ti6Al4V implants (**A**) or PCL-coated magnesium implants without previtalisation (**B**) or previtalised with murine osteoblasts (**C**) or mADMSCs (**D**). The magnesium implants appear to be dissolved after trichrome-masson-goldner staining (bo = bone tissue, c = connective tissue, el = elongated fixation strut, i = implant, sp = free space; scale bar = 1000 µm).

**Figure 16:**
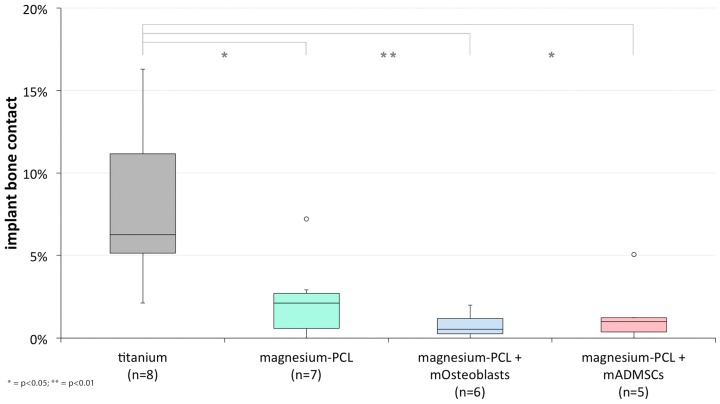
Implant bone contact (%) of uncoated titanium implants, non-previtalised Mg-PCL implants and Mg-PCL implants previtalised with either mOsteoblasts or mADMSCs (* = *p* < 0.05; ** = *p* < 0.01; whiskers = minimal and maximal values; horizontal line = median; circles = outliers).

Overall, the hybrid implants showed less bone contact in comparison to the titanium group. Within all implant treated groups there was found a tissue-free space between the elongated fixation struts and the bony anchoring area (Figure 15). While the titanium implants continued to adhere to the defect, resulting in a tighter connection to the calvarial bone, the hybrid implants were located at certain distances from the defect, causing the tissue-free space to enlarge. The surface of the hybrid implants facing the defect were surrounded by rather cellular connective tissue, but no mineralised bone could be found in direct contact to the implant regardless of the previtalisation status. The median value regarding the percentage implant bone contact for the titanium implant treated group was 6.25% (sd = 4.77%), while it was significantly lower for each Mg-PCL-treated group regardless of the previtalisation status (Figure 16; Mg-PCL: median = 2.11%, sd = 2.45%, *p* = 0.0128; Mg-PCL + mOsteoblasts: median= 0.53%, sd = 0.76%, *p* = 0.0032; Mg-PCL + mADMSCs: median = 1.00%, sd = 2.03%, *p* = 0.0154).

## 4. Discussion

The overall aim of this study was to evaluate the usage of PCL-coated and previtalised magnesium implants as an alternative to titanium for treating critically sized calvarial defects. While a balanced substitution rate of bone ingrowth and implant degradation were both desired and expected, the outcomes revealed an imbalance in favour of the latter. Namely, the analyses of the µCT scans showed a magnesium implant degradation of about 50% within 12 weeks, whereas there was only a significant bone formation within the first four weeks and only a slight increase during the remaining eight weeks of observation. The reason for the imbalance in implant bone substitution may lie either in an inadequate bone ingrowth or in a too rapid implant degradation. Judging from the outcomes of the bone volume (µCT) and bone area (histomorphometry) measurements, both untreated and titanium implant-treated mice showed a similar amount of newly built bone tissue within the defect as magnesium-treated ones. Histomorphometrical analyses of the amount of osteoid also revealed no significant differences between the experimental groups. Therefore, within this study the porous implant geometry did not show osteoconductive effects although it is reported to do so in several studies [46,47]. Since the magnesium implants mostly lost their adhesion to the defect due to mechanical manipulation and gas formation caused by the degradation process, possible osteoconductive effects may not have been able to take place within the defect area. Thus, the impact of the scaffold geometry could only be observed by evaluating the titanium implants. While Taniguchi et al. [48] showed high bone ingrowth for titanium scaffolds with 600 µm pore size, these effects could not be confirmed in our examinations. Rakhmatia et al. revealed that titanium meshes of 100 µm thickness cause high bone ingrowth within calvarial defects in rats [49]. While in that case the mesh thickness was smaller than the host’s calvaria, the implants used in our study were more than twice as thick as the murine skull bone. Therefore, an imbalance might have existed within the overall proportions between implant and host, resulting in the porous geometry not being accepted as such by the organism. The histomorphometrical analyses also showed an ingression of brain tissue within the lattice structure of the implants (Figure 4A, br), which might have hindered any other tissue, including bone, to infiltrate the implant pores. Since physiologically a consistent skull stabilises the intracranial pressure, as stated in the Monro-Kellie hypothesis [50], this phenomenon may be caused by the cerebral pressure itself. Therefore, it is possible that the pore spaces of the titanium implants had not been fully accessible since the day of surgery as the consistency of the skull was no longer existed. Although the implant geometry might not have positively influenced the bone ingrowth, the presence of an implant structure (titanium or magnesium) resulted in a significant increase in blood vessels within the osseous tissue on the defect treated skull side in comparison to non-implant-treated defects. As a minimal pore size of 400 µm is reported to benefit the ingrowth of blood vessels [51], the implant geometry may have induced this phenomenon. Additionally, a previtalisation of Mg-PCL implants resulted in a significant increase in blood vessels compared to titanium implants and, in terms of previtalisation with mADMSCs, to Mg-PCL implants without previtalisation. This can be traced back to the production of proangiogenic factors of both osteoblasts and mesenchymal stem cells and therefore their ability to enhance the healing process as reported in various studies [52,53,54,55]. When comparing both the defect (and possibly implant) treated right and the untreated left skull side a significant increase from left to right could be detected for both control groups. Since both angiogenesis and vasculogenesis are essential for bone healing this reflects the higher presence of regeneration processes around the defect [56]. Surprisingly, within all magnesium-based groups no significant differences between both skull sides regarding intraosteal blood vessels could be observed, while each of them featured significantly higher amounts of blood vessels compared to both control groups on the left skull side. Also, both µCT and histomorphometrical analyses revealed a significantly higher amount of newly formed bone tissue on the left side for magnesium-treated mice in comparison to control group 1 (mice without implants). These alterations in vascularisation and bone formation might have been caused by the degradation process of the magnesium implants: Since all implants were fixated at the left skull side the mechanical forces of the moving implants might have been conducted along the elongated struts to the bony anchoring area. Since according to Wolff’s law bone tissue structurally adapts to mechanical forces [57], the shifting magnesium scaffolds might have induced remodelling processes on the fixation site of the implants. Supporting this theory, Ikegame et al. discovered an osteoconductive effect of tensile stress within mouse calvarium organ cultures [58]. The correlation between the increase in bone volume/area and the magnesium corrosion would explain the absence of osseous remodeling on the left skull side within the titanium-treated group. The same applies to the significantly lesser amount of blood vessels. Against expectations, the overall bone formation within each experimental group was not sufficient enough to close the primarily set critical defect. However, it is remarkable that in all groups there was a significant increase in bone volume within the first 28 days, followed by only minimal growth during the subsequent 56 days of examination. The reason for this uncommon development may be found in the animals’ age. Although mice are deemed to be classified as adult at the age of 12 weeks [59], Kilborn et al. stated that the murine growth plate closes at the age of about five months [60]. Since the µCT examinations revealed a significant increase in bone volume not just at the treated but also at the untreated skull side for control group 1, possible physiological bone transformations may have overlapped with the first phase of µCT observation. The changes in bone volume on the untreated side mainly resulted from an increase in the calvarium thickness, which affirms the hypothesis that the mice, although commonly considered as adult, had probably not completely finished their natural bone growth at the time of surgery. Focusing again on the substitution index, the magnesium implants seemed to degrade about five to six times faster than bone ingrowth took place. Judging from the resulting disconnection of the implants from the defect, the degradation process might carry more weight on the substitution index in comparison to the bone ingrowth. Although the free space around the implants is not just caused by the degradation process—since it also occurred while using non-degradable titanium implants—the latter seemed to enlarge it to a certain extent. Therefore, the main amount of this space may represent so-called gas pockets such as those observed in various studies using magnesium-based implants [42,44,61]. Zhang et al. also reported that the gas pockets were enveloped by connective tissue [61]. This phenomenon could partly be confirmed since there were also strands of connective tissue underneath the hybrid implants while in the other direction potential gas pockets were demarcated by physiological connective tissue of the skin. As stated by Wong et al., the surface-to-volume ratio of magnesium implants may affect the occurrence of gas pockets since a larger implant surface makes the implant more vulnerable to degradation processes. It was also stated that the gas release rate of highly corrosive implants might surpass the maximum absorption rate of the host’s body, which then results in formation of gas pockets [32]. Due to its porous structure the surface-to-volume ratio of the implant geometry tested within our study was relatively high in comparison to the simple geometries used for long bone application. Therefore, it would also explain the absence of bone formation around the hybrid implants. In contrast, several studies reported proper bone formation in the presence of gas formation [62,63,64] but since these implants were always implanted in long bones the situation is not completely comparable to our study. Here, the main proportion of surrounding tissue was represented by skin and brain tissue so small changes in implant position may have disconnected it from the bony margins of the defect to an extent that may have made an interaction and therefore proper healing impossible. Although it is stated that PCL-coating enables a stable population of osteoblasts on coated magnesium implants in vitro [27], in our study it did not seem to contain the gas formation in dimensions that were needed for a proper fixation of the implants within the defect. As stated by Chen et al., the coating of pure magnesium with PCL leads to a specific interaction between the metal core and the polymer layer, resulting in a lowering of the corrosion resistance of the hybrid implant [29]. This may explain the rather fast degradation of the hybrid implants within our study compared to studies cited above which used alloyed magnesium as base material. Also, the mechanical manipulation of the implants during the previtalisation and implantation processes, although handled as gently as possible, may have caused scratches in the PCL-coating, resulting in an inconsistent polymer coating. Thus, created leaks may then have been passable for tissue fluid, resulting in a direct contact to the bare magnesium implant and therefore an accelerated degradation process. Several studies prove that PCL-coated magnesium implants show proper cytocompatibility to osteogenic cells in vitro [32,65] Although dissolved magnesium ions are stated to act osteoconductively by promoting osteoblast proliferation and therefore osteointegration [66] no comparable effect could be reported in our study. It is reported that the buffer capacity of surrounding fluids is crucial for the control of the effects of magnesium degradation on cell vitality and proliferation both in vitro and in vivo [67]. However, since within our study the hybrid implants were partly disconnected from surrounding tissue due to mechanical shifting and gas formation, the cells may have been exposed to the degradation process without any proper buffering system. Therefore, an uncontrolled change in pH value might have caused cell damage and death, which would explain the missing significant differences to non-previtalised samples in terms of osteointegration. When considering the increase in blood vessel formation within both previtalised hybrid implant groups the cells from the defect facing implant side may have also migrated to the surrounding natural bone structures locally, resulting in new formation of vascular tissue due to previously described mechanisms.

## 5. Conclusions

Regarding bone ingrowth inside the defect, the treatment with Mg-PCL implants resulted in neither significantly higher nor lower amounts of newly built bone tissue compared to the control groups. Previtalisation of those implants did not cause any significant changes in bone volume, area or amount of osteoid. Blood vessel formation around the defect was significantly higher within implant-treated mice, probably due to the positive effect of implant geometry and pore size, while a previtalisation of the Mg-PCL implants resulted in even significantly higher values. Magnesium implants degraded steadily but probably too fast, partially causing implants to lose their adhesion to the defect or to even be rejected by the animal. As a result, the percentage of implant bone contact was significantly lower compared to titanium and the substitution index for magnesium implants turned out to be five to six times higher than expected.

Overall, despite the insufficient degradation containment of the PCL-coating and the related effects thereof, magnesium-based implants proved to have no negative effects on bone regeneration. Furthermore, an additional previtalisation is favourable in terms of proper vascularisation. Although several refinements in corrosion resistance, fixation method and size wise implant host ratio are indicated for future examinations, this study still considers magnesium-based implants as being a promising alternative for treating critically sized calvarial defects. 

## Figures and Tables

**Table 1:** Experimental animal grouping.

Experimental Group Number	Surgery	Implant	Coating	Previtalisation
Control group 1	Defect	-	-	-
Control group 2	Defect	Titanium	-	-
Group 3	Defect	Magnesium	PCL	-
Group 4	Defect	Magnesium	PCL	Murine Osteoblasts
Group 5	Defect	Magnesium	PCL	Murine ADMSCs

## Supplementary Materials

The following are available online at https://figshare.com/s/e635c10c7571b0488cc9; Table S1: Median values and standard deviations for bone volume, implant volume, bone area, osteoid proportion and blood vessel observations. Figure S1: Exemplary µCT scan of a mouse treated with an uncoated titanium implant at day 0 (rostrocaudal view; i = implant with artefacts, s = skull); Figures S2–S29: Histological images of trichrome-masson-goldner stained thin sections of mice without implant (only defect; control group 1); Figures S30–S53: Histological images of trichrome-masson-goldner stained thin sections of mice treated with titanium implant (control group 2); Figures S54–S75: Histological images of trichrome-masson-goldner stained thin sections of mice treated with non-previtalised hybrid implant; Figures S76–S102: Histological images of trichrome-masson-goldner stained thin sections of mice treated with hybrid implant previtalised with murine osteoblasts; Figures S103–S124: Histological images of trichrome-masson-goldner stained thin sections of mice treated with hybrid implant previtalised with murine ADMSCs.
